# Land snail biogeography and endemism in south-eastern Africa: Implications for the Maputaland-Pondoland-Albany biodiversity hotspot

**DOI:** 10.1371/journal.pone.0248040

**Published:** 2021-03-04

**Authors:** Sandun J. Perera, David G. Herbert, Şerban Procheş, Syd Ramdhani

**Affiliations:** 1 School of Agricultural, Earth and Environmental Sciences, University of KwaZulu-Natal, Durban, South Africa; 2 KwaZulu-Natal Museum, Pietermaritzburg, South Africa; 3 School of Life Sciences, University of KwaZulu-Natal, Durban, South Africa; University of California, UNITED STATES

## Abstract

Invertebrates in general have long been underrepresented in studies on biodiversity, biogeography and conservation. Boundaries of biodiversity hotspots are often delimited intuitively based on floristic endemism and have seldom been empirically tested using actual species distributions, and especially invertebrates. Here we analyse the zoogeography of terrestrial malacofauna from south-eastern Africa (SEA), proposing the first mollusc-based numerical regionalisation for the area. We also discuss patterns and centres of land snail endemism, thence assessing the importance and the delimitation of the Maputaland-Pondoland-Albany (MPA) biodiversity hotspot for their conservation. An incidence matrix compiled for relatively well-collected lineages of land snails and slugs (73 taxa in twelve genera) in 40 *a priori* operational geographic units was subjected to (a) phenetic agglomerative hierarchical clustering using unweighted pair-group method with arithmetic means (UPGMA), (b) parsimony analysis of endemicity (PAE) and biotic element analysis (BEA). Fulfilling the primary objective of our study, the UPGMA dendrogram provided a hierarchical regionalisation and identified five centres of molluscan endemism for SEA, while the PAE confirmed six areas of endemism, also supported by the BEA. The regionalisation recovers a zoogeographic province similar to the MPA hotspot, but with a conspicuous westward extension into Knysna (towards the Cape). The MPA province, centres and areas of endemism, biotic elements as well as the spatial patterns of species richness and endemism, support the MPA hotspot, but suggest further extensions resulting in a greater MPA region of land snail endemism (also with a northward extension into sky islands—Soutpansberg and Wolkberg), similar to that noted for vertebrates. The greater MPA region provides a more robustly defined region of conservation concern, with centres of endemism serving as local conservation priorities.

## Introduction

Terrestrial molluscs (Mollusca: Gastropoda; hereafter land snails) with a global species richness estimated at over 25,000 [1, 2] are not distributed evenly across the globe. Although several island land snail faunas are reported to have exceptional species richness and high degrees of endemism [3, 4], and are thus recognized as hotspots for molluscan conservation, similar continental hotspots are not widely recognized. Global biodiversity hotspots [5, 6] have been delimited based on floristic endemism, often with the assumption of congruent distribution patterns for animals, but such congruence of endemism between plants and land snails is yet to be well established. Invertebrates in general have long been underrepresented in studies on biodiversity, biogeography and conservation, primarily because much of their diversity is still to be documented, while their taxonomy is often not sufficiently robust and the knowledge on their distribution is incomplete [5, 7–11]. Sub-Saharan Africa has a rich terrestrial malacofauna with an estimated 6,000 species [12]. Land snails in South Africa (SA) are taxonomically better resolved and distributionally better understood compared to many other groups of invertebrates, at least in eastern SA [13]. Over 650 land snail species are recorded for southern Africa [14], while 525 species (with *c*. 90% endemism) occur in SA alone [15]. However, their biogeography has never been systematically studied in southern Africa, except for qualitative studies [13, 14]. The terrestrial malacofauna of eastern SA includes relict Gondwanan lineages and speciose radiations of more recent origin exhibiting high levels of local endemicity [13]. The indigenous land snail fauna of eastern SA includes well over 270 species, with at least 173 (63%) species being endemic or near-endemic [13]. With high levels of diversity and endemism, land snails make an excellent indicator group to start exploring the invertebrate zoogeography of south-eastern Africa (SEA). Narrow-range distribution patterns commonly observed in land snails, stemming from their low dispersal ability, make them a suitable candidate group to study regional endemicity and historical biogeography [4, 16, 17], and to determine priority areas for conservation [18, 19], at least for the best studied groups.

Boundaries of biodiversity hotspots have seldom been empirically tested on actual species distribution ranges and not for endemic invertebrate fauna. The Maputaland-Pondoland-Albany (MPA) biodiversity hotspot of SEA has been delimited based on intuitive phytogeographical units such as the Maputaland-Pondoland region and the Albany centre of floristic endemism [20]. Possessing more than 1,900 species of endemic higher plants, this hotspot spans the eastern coast of southern Africa from Maputo in Mozambique to Port Elizabeth in Eastern Cape province of SA and extends inland to reach an altitude of 1,800 m a.s.l. along the south-eastern portion of the Great Escarpment [21]. The MPA hotspot has recently been further explored in terms of its vertebrate endemism, documenting 62 endemic and 60 near-endemic species, and further recognizing a greater MPA region of vertebrate endemism [22–24]. The greater MPA region with 166 endemic vertebrate species is an expansion of the MPA hotspot’s area by 59%, to include 168% more endemic species, indicating a higher animal endemicity in areas adjacent to the original flora-based hotspot [24].

Here we numerically analyse the zoogeography of the terrestrial malacofauna in SEA for the first time, also assessing the importance and the delimitation of the MPA biodiversity hotspot for land snail conservation. This is achieved through numerical biogeographical analyses and mapping of species richness and endemism patterns followed by comparison of the results with the current boundary of the MPA hotspot. Biogeographical analyses are presented with three numerical approaches, our primary objective being a phenetic agglomerative hierarchical clustering of incidence data to propose a land snail-based biogeographical regionalisation and secondly to identify centres of land snail endemism, for which consensus and congruency is sought from two alternative approaches, a parsimony analysis of endemicity and a biotic element analysis.

## Materials and methods

### Study area & operational geographic units

**Fig 1:**
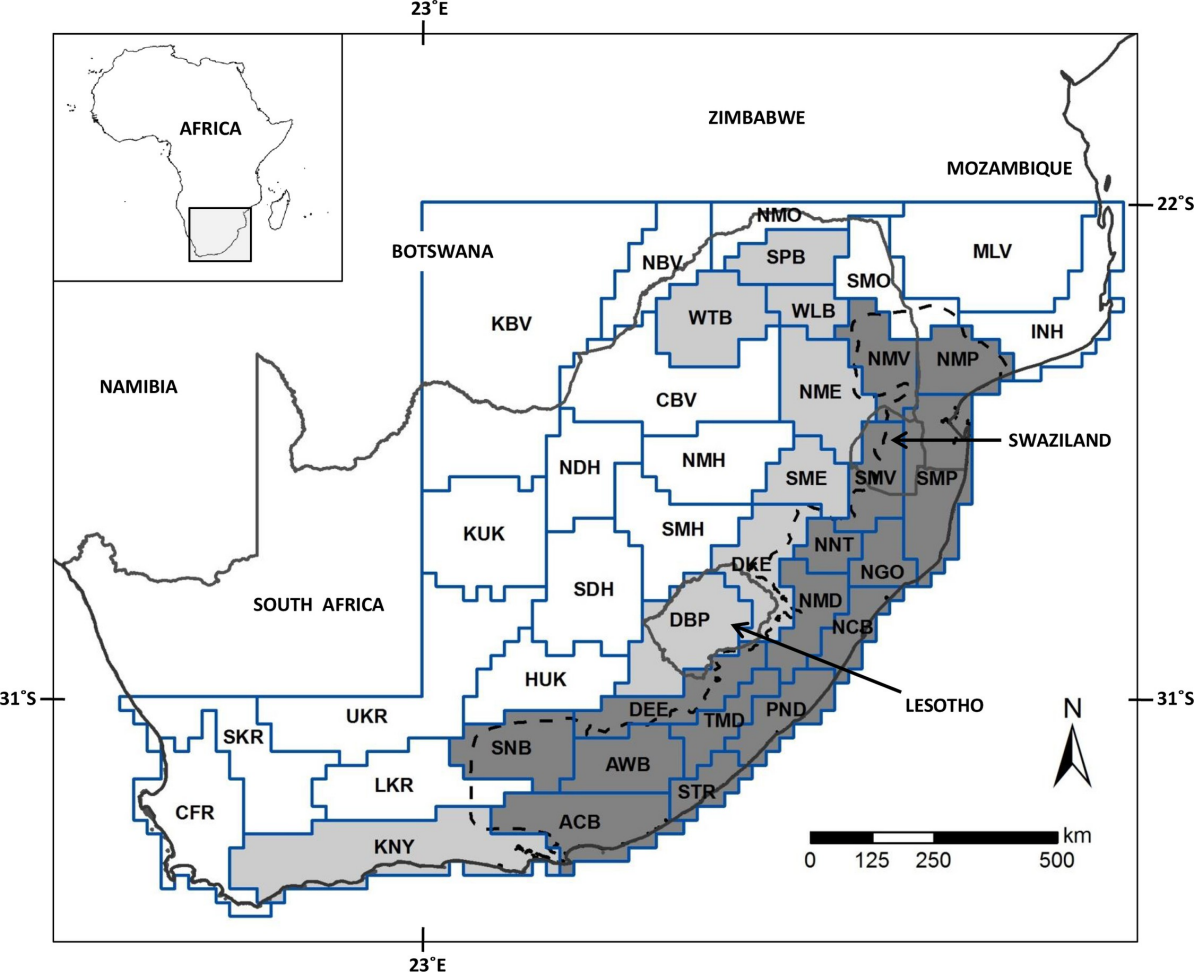
The study area showing the Operational Geographic Units (OGUs). The Maputaland-Pondoland-Albany (MPA) biodiversity hotspot is demarcated by the dashed line and the OGUs considered here as MPA units are indicated in dark grey, while the greater MPA region of vertebrate endemism [24] is in light grey. The OGUs are labelled as: ACB–Albany coastal belt, AWB–Amatola-Winterberg, CBV–central Bushveld, CFR–Cape Floristic Region except Knysna transition zone, DBP–Drakensberg plateau, DEE–Drakensberg-Eastern Cape escarpment, DKE–Drakensberg-KwaZulu-Natal escarpment, HUK–Highveld-upper Karoo, INH–Inhambane, KBV–Kalahari-Bushveld, KNY–Knysna transition zone (shared between the Cape Floristic Region and the greater MPA region), KUK–Kalahari-upper Karoo, LKR–lower Karoo, MLV–Mozambique lowveld, NBV–northern Bushveld, NCB–Natal coastal belt, NDH–northern dry Highveld, NGO–Ngoye, NMD–Natal Midlands, NME–northern Mpumalanga escarpment, NMH–northern mesic Highveld, NMO–northern mopane, NMP–northern Maputaland, NMV–northern Middleveld, NNT–northern Natal, PND–Pondoland, SDH–southern dry Highveld, SKR–Succulent Karoo, SME–southern Mpumalanga escarpment, SMH–southern mesic Highveld, SMO–southern mopane, SMP–southern Maputaland, SMV–southern Middleveld, SNB–Sneeuberg, SPB–Soutpansberg, STR–southern Transkei coastal belt, TMD–Transkei Midlands, UKR–upper Karoo, WLB–Wolkberg, WTB–Waterberg. See text for further details. The MPA hotspot boundary is reprinted from Hoffman et al. [26] (public domain).

The study area is southern Africa, south-east of 22°S and 23°E and south-west of 31°S and 23°E (Fig 1). This includes the MPA hotspot, the greater MPA region of vertebrate endemism as well as the SEA zoogeographical dominion for vertebrates [24]. Furthermore, the entire Cape Floristic Region (CFR) biodiversity hotspot was also included in order to clarify the uncertain western limits of the SEA dominion.

Land snail distribution data for the area could still be too incomplete for a numerical zoogeographical analysis at a fine-grain such as the quarter-degree-squares (QDSs) scale. Therefore, a set of coarse-scale eco-geographical units (zoogeographical units for SEA [22], redrawn according to QDS borders; see Fig 1) were used as the operational geographic units (OGUs) for the present analyses. The use of these natural OGUs (delimited through qualitative overlaying or range maps in identification of biochoria for endemic vertebrates; see Perera et al. [22] for more details) for recording the incidence of selected land snail genera (a) avoids the caveats of incompleteness of distribution data at a finer grain of analysis, and (b) retains the natural geographical boundaries of the resulting zoogeographic entities that would have been distorted had a coarse equal-area OGU (e.g. 1° or 2° grid cells) been used. The OGUs for the western section of the study area, beyond Knysna were defined based on the CFR and Succulent Karoo biodiversity hotspots [5, 6], and the bioregions of SA [25], resulting in a total of 40 *a priori* OGUs.

### Species incidence data matrix

Twelve genera (monophyletic lineages) of land snails and slugs were selected for the study, all of which have recent species-level taxonomic revisions [2, 27–37]. We included all species described to date in each genus from the study area, with a few exceptions as detailed below. Hence the analysis included 73 taxa, comprising 70 species and three species complexes of two species each. These genera were selected due to, (a) their high endemicity within the study area, (b) they are comparatively well sampled and collected within the study area, and (c) they have a relatively recent well resolved and robust taxonomy. The selected genera represent three families, *viz*. Bothriembryontidae, Rhytididae and Urocyclidae. All genera occurring within the study area were included from the first two families, while the family Urocyclidae, was represented by the most speciose monophyletic clade of the family *Sheldonia sensu lato* [13, 27, 28], of which the taxonomy was recently resolved with morphological and molecular support recognising five genera from the study area; viz. *Kerkophorus*, *Microkerkus*, *Ptilototheca*, *Selatodryas* and *Sheldonia sensu stricto* [28, 29]. The family Bothriembryontidae is represented in the area by three species in the genus *Prestonella* of the newly validated subfamily Prestonellinae, of which only two species were included in this analysis, excluding *P*. *quadingensis* due to the unavailability of distribution data except the type locality and absence of any specimens or recent observations; [30–33, DGH pers. obs.]. Land snails and slugs of the family Rhytididae are represented in the area by nine species of the hunter slug genus *Chlamydephorus* [13], three genera of larger cannibal snails: *Afrorhytida*, *Capitina*, *Natalina* (four, two and six species, respectively) [34, 35] and two genera of dwarf cannibal snails: *Nata* (six species) and the monotypic genus *Natella* [36, 37], of which all species were included in the current analysis except for treating *Nata erugata* and *N*. *vernicosa* as a single entity. While the delimitation of these two species relied heavily on molecular data, *Nata erugata-vernicosa* is believed to represent a species complex with additional unresolved taxonomic issues and hence ambiguities exist in their distribution records [36, DGH pers. obs.]. The family Urocyclidae is represented in the analyses by the genera *Kerkophorus*, *Microkerkus*, *Ptilototheca*, *Selatodryas* and *Sheldonia*. All species of *Microkerkus* (10), *Ptilototheca* (one) and *Sheldonia* (13) found in the study area were included, while *Sheldonia hewitti* was considered as a synonym of *S*. *aloicola* [2, DGH pers. obs.]. The genus *Kerkophorus* was represented in the area by 20 species and is included in the analysis as 18 definitive species plus the *Kerkophorus piperatus-vittarubra* complex, while *K*. *phaedimus* was considered to be a synonym of *K*. *corneus* [2]. The *K*. *piperatus-vittarubra* complex was treated as such due to the difficulty in morphologically separating taxa at some locations due to their close relatedness and even a possible hybrid population in Nkandla [28]. The genus *Selatodryas* being an endemic lineage in the study area with a narrow geographical range and represented by two morphologically similar species that can only be reliably identified through dissection of the genital tract was also included in the analysis as the *Selatodryas luteosoma-roseosoma* complex. These three species complexes, with the known localities for the two described species in each combined, are treated as three species hereafter (given with their specific epithets combined by a hyphen).

The species incidence matrix was prepared for the 73 species above by scoring their presence/absence (1/0) in 40 *a priori* OGUs (S1 Appendix). A species was recorded as present for a given OGU, even with a single occupied QDS, as long as this QDS was not along the margin of the OGU. A species occupying only marginal QDSs for a given OGU was scored as present only if the species occupied more than 10% of the OGU. A species occupying marginal QDSs with <10% coverage of the OGU was considered present only when the species was absent in the neighbouring OGU, or the relevant QDS was in the coastal margin of its range. Species distribution data were sourced from the KwaZulu-Natal Museum Mollusca database (more than 2300 records, with field visits to all OGUs), after removal of dubious records.

### Data analyses

#### Zoogeographical regionalisation

Numerical analysis of the land snail incidence matrix, following the phenetic approach [38] of agglomerative hierarchical clustering of OGUs was used to fulfil the primary objective of the study, i.e. to propose a land snail based biogeographical regionalisation (see Kreft & Jetz [39] and Morrone [40] for recent reviews on methods). The Jaccard’s coefficient of similarity was used to compare each OGU with every other based on their respective species assemblages [41–44], while the unweighted pair-group method using arithmetic averages (UPGMA) algorithm was used to convert the similarity matrix into a distance based dendrogram [39, 43–46]. Mouillot et al. [47] and Perera et al. [24] provides a discussion on the above methods. The analyses were conducted using FreeTree *ver*. 0.9.1.50 [48]. The resulting dendrograms were visualised using TreeView *ver*. 1.6.6. [49]. The subregional hierarchy of zoogeographical entities *i*.*e*., dominions, subdominions, provinces, subprovinces and districts [40, 50–52] (see Perera et al [24] for more details) were determined based on phenon lines [38, 53] placed on the dendrogram in order to generate geographically contiguous clusters of OGUs, while OGUs that were not placed in such geographically contiguous clusters were dissolved in to the geographically nearest and ecologically closest biogeographical entity derived from the same dendrogram [23, 24].

#### Centres of endemism

Centres of endemism (COEs: areas where endemic species concentrate, usually having more endemics in comparison to the surrounding areas) [54–56], were also derived from the UPGMA cluster dendrogram. The COEs and centres of narrow endemism (CONEs) are presented based on decreasing range size and number of endemics: (a) COEs: clusters identified above as biogeographical districts that harbour four or more endemic land snail species and (b) CONEs: single OGUs or geographically narrow clusters of OGUs defined by a higher similarity than the districts, harbouring two or more narrowly endemic species. Characteristic and narrow endemics for each COE were identified following Williams et al. [54], Crisp et al. [57] and Procheş & Ramdhani [43]. Characteristic endemics are defined here as species occupying more than two-thirds of OGUs in the centre, and/or distributed over more than half of the centre’s extent, hence whose range edges roughly coincide with the boundary of the centre, while the narrow endemics occupy much narrower ranges within the COE.

#### Areas of endemism and biotic elements

Areas of endemism (AOEs: areas with congruent distribution of at least two species of restricted range) [55, 58] (see Perera et al. [24] for a discussion on AOEs and COEs) were identified through the parsimony analysis of endemicity (PAE) [59]. However, we did not attempt a regionalisation based on the PAE as done is some studies [24, 60, 61] as the resulting area cladogram revealed only five clusters, although not hierarchically structured into larger AOEs, among which only four harboured at least two endemic species with sympatry, in addition to two terminal OGUs each with two narrow endemics with congruent ranges.

The PAE was conducted using PAUP* 4.0b10 [62], where a full heuristic search was performed with tree bisection-reconnection (TBR) branch swapping, after the species present or absent in all OGUs (similar to constant characters in phylogenetics) and species found only in a single OGUs (i.e. parsimony uninformative characters; autapomorphies in phylogenetics) were excluded. A strict consensus tree was constructed from 1000 most parsimonious trees. Geographically contiguous clusters of OGUs in the strict consensus tree harbouring at least two endemic species with sympatry were recognized as AOEs. In the description of PAE methodology Morrone [59] recommends to “draw quadrats on a map of the region to be analysed, considering quadrats only where at least one locality of one species exists” (p. 438), i.e. to remove any OGUs with zero presence of species in our dataset, and as such these OGUs are similar to outgroups and the parsimony analysis effectively remove multiple outgroups. Furthermore, during the parsimony analysis in phylogenetics, autapomorphies are removed from the dataset as these are considered uninformative. Similarly, during the PAE in biogeography, parsimony uninformative species (which are restricted to a single OGU) are removed from the analysis. However, such narrowly endemic species that are of conservation importance were included and considered in the delimitation of COEs.

However, the PAE area cladogram did not show hierarchical structuring so we further tested our AOEs for consensus and congruency with those recovered from a biotic element analysis (BEA). The BEA proposed by Hausdorf & Hennig [63] is an alternative to AOEs, in identification of vicariant events during the history of an area. We performed a BEA which included a parametric bootstrap test for clustering of distribution areas [64] using the programme PRABCLUS in R studio [65].

#### Spatial patterns of land snail richness and endemism

The following spatially-based quantitative measures of land snail richness and endemism were calculated for each OGU using the species incidence matrix: (a) land snail species richness, (b) SEA dominion endemism, (c) narrow endemism, and (d) weighted endemism (WE). The measure (a) was calculated for the entire study area, while the latter parameters (b‒d) were calculated only for the SEA dominion, delineated by the regionalisation above. Here a narrow endemic species was defined as one restricted to a single OGU, a CONE or occupying less than half the range of a COE recognized above. Based on this definition, *Prestonella nuptialis*, *Afrorhytida trimeni* and *Selatodryas luteosoma-roseosoma* were considered narrow endemic even though they occupy two OGUs each, on the basis that the OGUs concerned are narrow in extent and they each occupy less than half the area of the OGUs combined. On the other hand, *Afrorhytida kraussi* and *Sheldonia phytostylus* although present only in the Knysna OGU were not considered as narrow endemics on the basis that the Knysna OGU was too broad and the species are distributed in more than half of its extent. The WE of each OGU was calculated as the sum of the reciprocals of the total number of OGUs in which each species of the respective OGU was found [56]. Further, the WE was corrected for the species richness in respective OGUs (= corrected WE [56]). Values obtained for all measures were normalized to stand between 0 and 1. Each of the above parameters were mapped using ArcMap10.1 [66] with a graduated grey scale for a maximum of five classes determined by natural breaks calculated using Jenk’s optimisation, so that the patterns inherent in the data are best revealed.

## Results

### Biogeography of land snails in south-eastern Africa

**Fig 2:**
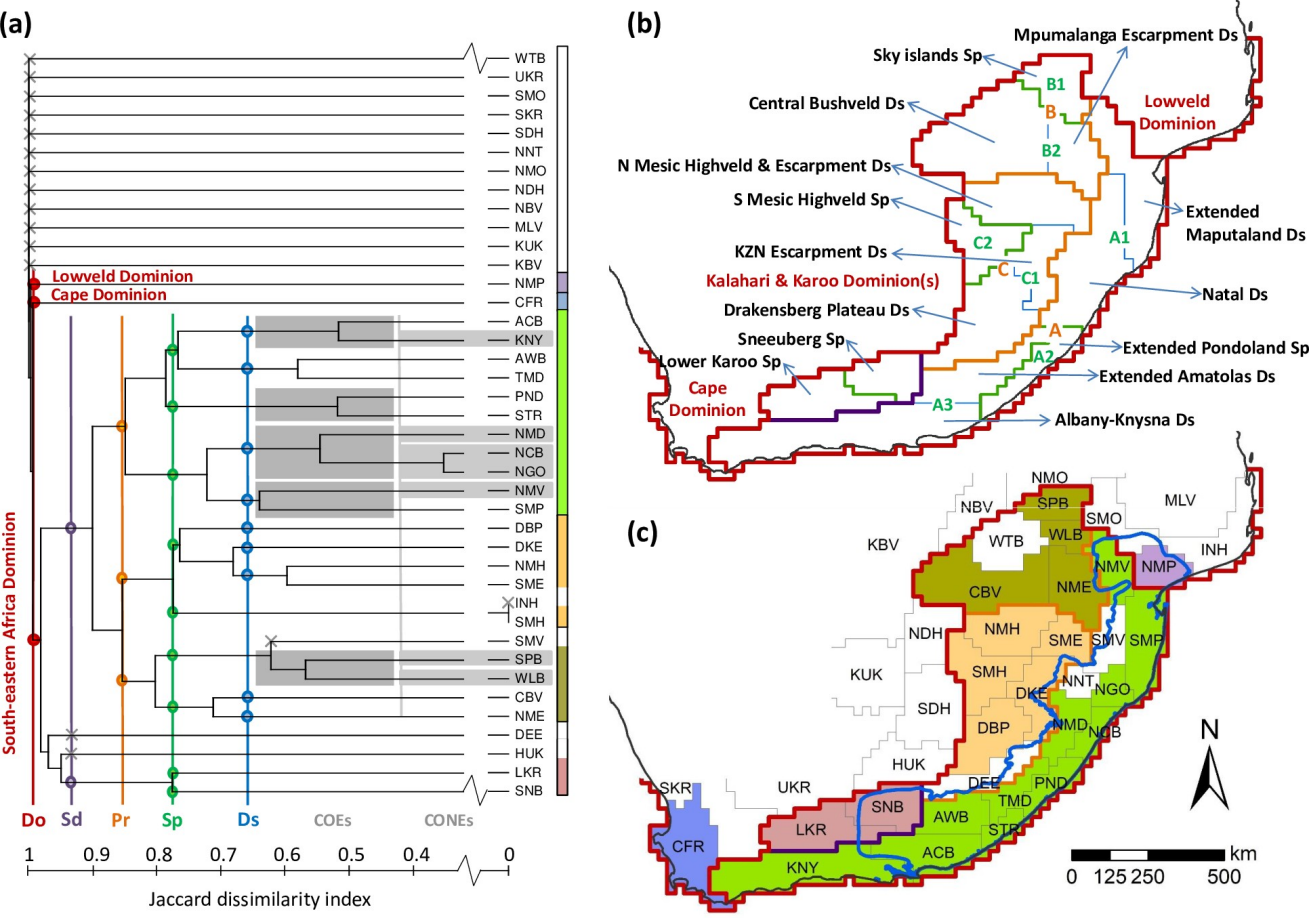
Proposed zoogeographical regionalisation for land snails of south-eastern Africa. **(a)** Dendrogram of hierarchical relationships between operational geographic units (OGUs), based on UPGMA clustering with Jaccard’s coefficient of similarity. The hierarchy of zoogeographical entities were established using phenon lines of increasing similarity: Do = dominion; Sd = subdominions; Pr = provinces; Sp = subprovinces and Ds = districts. Centres of endemism (dark grey) and centres of narrow endemism (light grey) are also recognized from the same dendrogram, and mapped in Fig 4. Three letter codes for OGU names follow Fig 1. **(b)** The proposed hierarchical zoogeographical regionalisation of the South-Eastern Africa (SEA) dominion (thick dark red boundary; Lowveld and Cape dominions respectively in north-east and south-west are also indicated). The coloured phenon lines in panel (a) defines subdominions, provinces, subprovinces and districts of the SEA dominion, listed in Table 1, with respective codes for provinces (A-C), subprovinces (A1, A2, etc.). Entities labelled on the map are subprovinces (Sp) and districts (Ds); S = South, N = North and KZN = KwaZulu-Natal. **(c)** Malacofaunal provinces within the two subdominions of SEA dominion (separated by thick purple boundary): MPA-Highveld subdominion with three provinces—MPA-Knysna province (light green), Highveld province (pale orange) and Bushveld province (olive green); and southern escarpment subdominion with no subordinate provinces (pale maroon). OGUs shown here in white (and marked with **×** on the dendrogram) do not form geographically contiguous clusters, and were hence dissolved into the geographically nearest and ecologically closest biogeographical entity (see **discussion** for more details). MPA hotspot boundary is reprinted from Hoffman et al. [26] (public domain) in blue to show its recovery as a valid zoogeographical entity (MPA-Knysna province), despite an extension towards Knysna in south-west.

**Fig 3:**
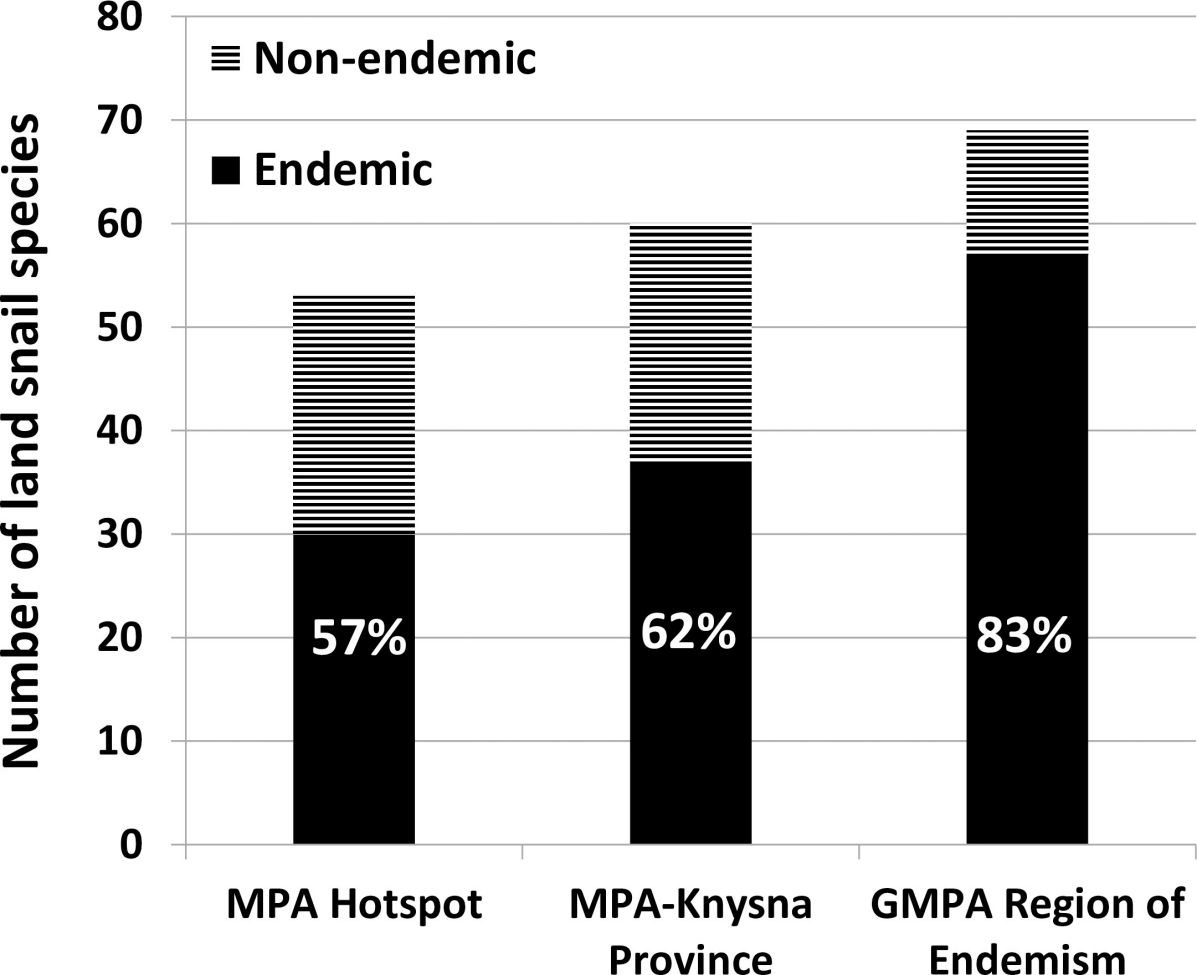
Land snail species richness, endemism and percentage endemicity within the MPA hotspot, MPA-Knysna province and the greater MPA region of land snail endemism.

The first ever numerical regionalisation for land snails in south-eastern Africa is presented in Fig 2 and Table 1. An initial division of the UPGMA dendrogram (Fig 2A) separates a very clear south-east African cluster. It is placed within the continental/global zoogeographical context, as the SEA dominion [24] within the southern African subregion (given as southern African region in Linder et al. [45]) of the Afrotropical region (as in Procheş & Ramdhani [43], given as Afrotropical realm in Holt et al [46]). Subordinate subdominions, provinces, subprovinces and districts are delimited within the SEA dominion (Fig 2B, 2C and Table 1). The dendrogram recovers a land snail based biogeographical province similar to the MPA hotspot, despite extending westward from Albany to include the Knysna area. The MPA-Knysna province consequently defined in this study has 67% congruence to the MPA hotspot in terms of OGU coverage (Fig 2B). The MPA-Knysna province harbours 37 endemic species (61.7% endemicity; Table 2) in the selected land snail genera, which is higher than the 30 endemics (56.6% endemicity; Table 2) within the MPA hotspot (Fig 3).

**Table 1:** Proposed regionalisation hierarchy for land snail zoogeography in south-eastern Africa (SEA).

Dominion	Subdominion	Provinces		Subprovinces		Districts
South–Eastern Africa	Maputaland-Pondoland-Albany-Highveld	A	Maputaland-Pondoland-Albany-Knysna	A1	Maputaland-Natal	Extended Maputaland
						Natal
				A2	Extended Pondoland	
				A3	Extended Albany	Extended Amatolas
						Albany-Knysna
		B	Bushveld	B1	Sky Islands (Soutpansberg-Wolkberg)	
				B2	Bushveld-Mpumalanga Escarpment	Central Bushveld
						Mpumalanga Escarpment
		C	Highveld	C1	Northern Mesic Highveld & Escarpment	Northern Mesic Highveld & Mpumalanga Escarpment
						KwaZulu-Natal Escarpment
						Drakensberg Plateau
				C2	Southern Mesic Highveld	
	Southern Escarpment				Sneeuberg	
					Lower Karoo	

The SEA dominion is placed within the southern Africa subregion of the Afrotropical region (see Fig 2A for the dendrogram, 2B for the map, and the text for further details).

**Table 2:** Land snail species selected for the study (n = 73; representing 12 genera and three families) and their endemism within the Maputaland-Pondoland-Albany (MPA) biodiversity hotspot, the MPA-Knysna province, the greater MPA region of land snail endemism, and the south-eastern Africa (SEA) dominion.

Family	Species	Endemic to				
		MPA-hotspot	MPA-Knysna province	Greater MPA region of land snail endemism	South-eastern Africa dominion	The study area
Bothriembryontidae	*Prestonella bowkeri*				√	√
Bothriembryontidae	*Prestonella nuptialis* ^NE^	√		√	√	√
Rhytididae	*Chlamydephorus bruggeni* ^NE^	√	√	√	√	√
Rhytididae	*Chlamydephorus burnupi*	* *	* *	√	√	√
Rhytididae	*Chlamydephorus dimidius*	√	√	√	√	√
Rhytididae	*Chlamydephorus gibbonsi*	√	√	√	√	√
Rhytididae	*Chlamydephorus lawrencei*			√	√	√
Rhytididae	*Chlamydephorus parva* ^NE^	√	√	√	√	√
Rhytididae	*Chlamydephorus purcelli*					√
Rhytididae	*Chlamydephorus sexangulus*		√	√	√	√
Rhytididae	*Chlamydephorus watsoni*	√	√	√	√	√
Rhytididae	*Afrorhytida burseyae*	√	* *	√	√	√
Rhytididae	*Afrorhytida knysnaensis*	√		√	√	√
Rhytididae	*Afrorhytida kraussi*		√	√	√	√
Rhytididae	*Afrorhytida trimeni* ^NE^	√	√	√	√	√
Rhytididae	*Capitina calcicola*					√
Rhytididae	*Capitina schaerfiae* ^NE^		√	√	√	√
Rhytididae	*Nata aequiplicata* ^NE^	√	√	√	√	√
Rhytididae	*Nata dumeticola*					Near-endemic
Rhytididae	*Nata tarachodes*					√
Rhytididae	*Nata vernicosa-erugata*					√
Rhytididae	*Nata watsoni*	√		√	√	√
Rhytididae	*Natalina beyrichi*	√	√	√	√	√
Rhytididae	*Natalina cafra*	√	√	√	√	√
Rhytididae	*Natalina inhluzana* ^NE^	√	√	√	√	√
Rhytididae	*Natalina quekettiana*	* *	* *	√	√	√
Rhytididae	*Natalina reenenensis* ^NE^	√	√	√	√	√
Rhytididae	*Natalina wesseliana*					√
Rhytididae	*Natella viridescens*	* *	* *	* *	√	√
Urocyclidae	*Kerkophorus ampliatus* ^NE^	√	√	√	√	√
Urocyclidae	*Kerkophorus bicolor* ^NE^		√	√	√	√
Urocyclidae	*Kerkophorus cingulatus*		√	√	√	√
Urocyclidae	*Kerkophorus corneus*		* *	√	√	√
Urocyclidae	*Kerkophorus inunctus*	√	√	√	√	√
Urocyclidae	*Kerkophorus knysnaensis*		√	√	√	√
Urocyclidae	*Kerkophorus melvilli* ^NE^	√	√	√	√	√
Urocyclidae	*Kerkophorus perfragilis* ^**NE**^		* *	√	√	√
Urocyclidae	*Kerkophorus perlevis*		√	√	√	√
Urocyclidae	*Kerkophorus piperatus-vittarubra*		* *	√	√	√
Urocyclidae	*Kerkophorus poeppigii*	* *	* *	* *	√	√
Urocyclidae	*Kerkophorus pumilio* ^NE^		* *	√	√	√
Urocyclidae	*Kerkophorus puzeyi* ^NE^	√	√	√	√	√
Urocyclidae	*Kerkophorus russofulgens* ^NE^	√	√	√	√	√
Urocyclidae	*Kerkophorus scrobicolus* ^NE^	√	√	√	√	√
Urocyclidae	*Kerkophorus terrestris* ^NE^	√	√	√	√	√
Urocyclidae	*Kerkophorus vandenbroeckii* ^NE^		* *	√	√	√
Urocyclidae	*Kerkophorus vitalis*	√	√	√	√	√
Urocyclidae	*Kerkophorus zonamydrus*		√	√	√	√
Urocyclidae	*Microkerkus arnotti*					√
Urocyclidae	*Microkerkus burnupi* ^NE^		√	√	√	√
Urocyclidae	*Microkerkus chrysoprasinus*	* *	* *	√	√	√
Urocyclidae	*Microkerkus fuscicolor*	* *	* *	* *	√	√
Urocyclidae	*Microkerkus leucospira*	√	√	√	√	√
Urocyclidae	*Microkerkus maseruensis* ^NE^	* *	* *	√	√	√
Urocyclidae	*Microkerkus pondoensis* ^NE^	√	√	√	√	√
Urocyclidae	*Microkerkus sibaya* ^NE^	√	√	√	√	√
Urocyclidae	*Microkerkus symmetricus*	* *	* *	* *	√	√
Urocyclidae	*Microkerkus transvaalensis*		* *	√	√	√
Urocyclidae	*Ptilototheca soutpansbergensis* ^NE^		* *	√	√	√
Urocyclidae	*Selatodryas luteosoma-roseosoma* ^NE^	√	* *	√	√	√
Urocyclidae	*Sheldonia aloicola*	√	√	√	√	√
Urocyclidae	*Sheldonia asthenes*			√	√	√
Urocyclidae	*Sheldonia caledonensis*					√
Urocyclidae	*Sheldonia capsula*					√
Urocyclidae	*Sheldonia cotyledonis*					√
Urocyclidae	*Sheldonia crawfordi*			√	√	√
Urocyclidae	*Sheldonia fingolandensis* ^NE^	√	√	√	√	√
Urocyclidae	*Sheldonia hudsoniae*		√	√	√	√
Urocyclidae	*Sheldonia monsmaripi* ^NE^	√	√	√	√	√
Urocyclidae	*Sheldonia natalensis*		√	√	√	√
Urocyclidae	*Sheldonia phytostylus*		√	√	√	√
Urocyclidae	*Sheldonia trotteriana*				√	√
Urocyclidae	*Sheldonia wolkbergensis* ^NE^		* *	√	√	√
	**Total endemics**	30	37	57	63	72
	**Total species**	53	60	69	70	73
	**Species endemicity (%)**	**56.6**	**61.7**	**82.6**	90.0	98.6

Species endemicity is given for each geographical entity.

^**NE**^ denotes species considered as narrow endemics within the SEA dominion (see text for criteria).

### Land snail endemism in south-eastern Africa

**Fig 4:**
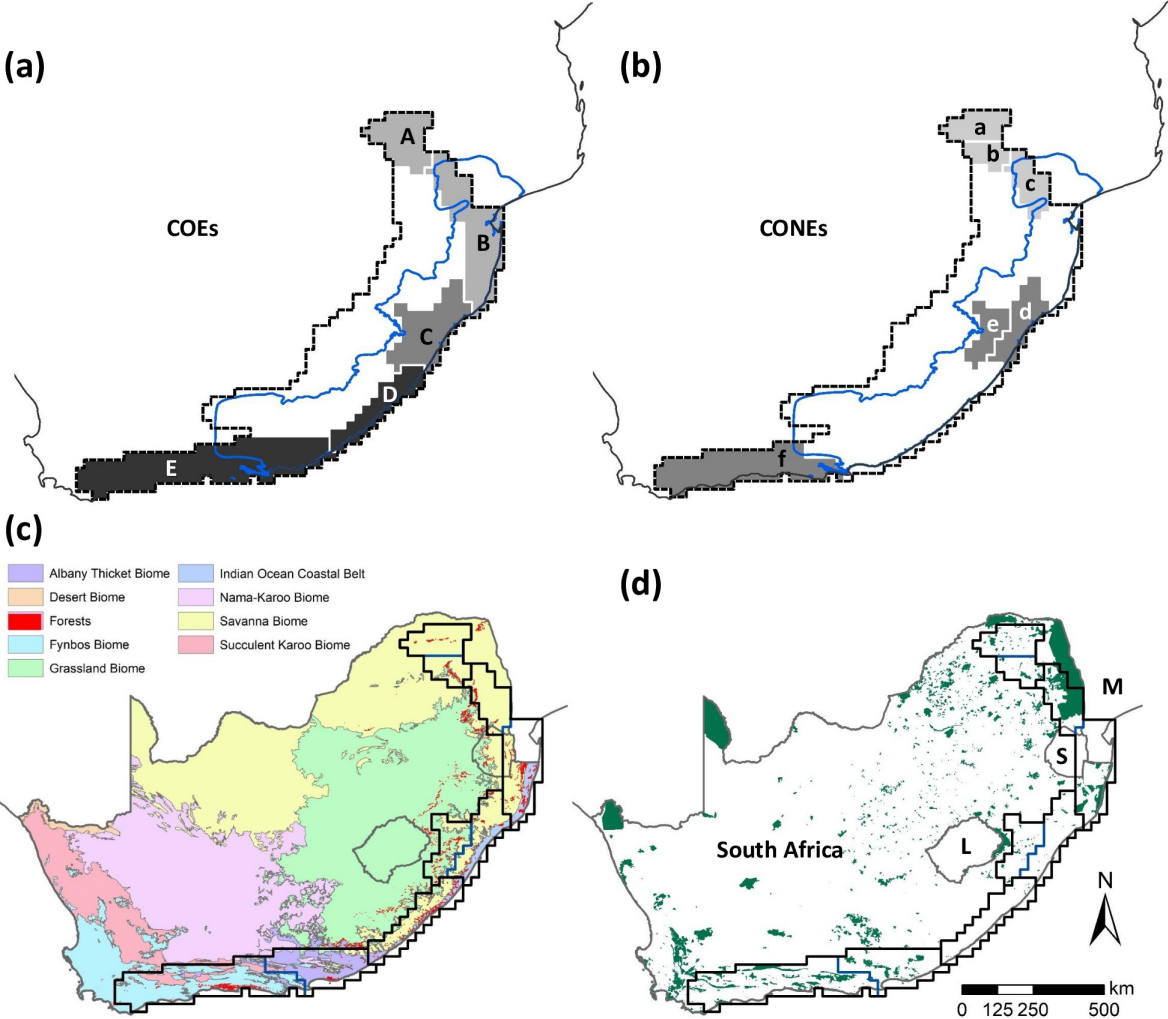
Centres of endemism (COEs) and centres of narrow endemism (CONEs) for land snails of south-eastern Africa derived from the phenetic cluster dendrogram (Fig 2A). Centres are shaded on a grey scale based on number of endemics, given below for each in brackets (darker shades denote high endemism). See Table 3 for lists of endemic species. **(a)** COEs: A–Sky islands (4), B–Extended Maputaland (4), C–Natal (6), D—Extended Pondoland (7), and E—Albany-Knysna (7). **(b)** CONEs: a—Soutpansberg (2), b—Wolkberg (2), c—Northern Middleveld (2), d—Natal coastal belt-Ngoye (3), e—Natal Midlands, and f—Knysna (3). The whole (blue) line indicates the Maputaland-Pondoland-Albany (MPA) hotspot boundary while the dashed (black) line delimits the greater MPA region of land snail endemism. COEs and CONEs are spatially compared with **(c)** South African biomes and **(d)** the protected area network of South Africa (SA); protected areas in Lesotho (L), Swaziland (S) and Mozambique (M) are not included. The MPA hotspot boundary, biomes and protected areas of SA are reprinted from Hoffman et al. [26], Open Knowledge Foundation [67] and the Department of Environmental Affairs, Forestry and Fisheries [68] (public domain), respectively.

Five COEs and six CONEs derived from the UPGMA dendrogram (Fig 2A) and are illustrated in Fig 4A and 4B, respectively.

**Table 3:** Range-restricted species endemic to centres of endemism (COEs), centres of narrow endemism (CONEs) and areas of endemism (AOEs) within the south-eastern Africa dominion (see Fig 4), also showing their nested hierarchy, verification as and areas of endemism (AOEs) and corresponding biotic elements (see text for details).

COEs ^a^		CONEs ^d^	
**also verified as AOEs ^b^		**also verified as AOEs ^b^	
and supported by biotic elements ^c^			
COE (no. of endemics)	List of centre endemics * and, species in the same biotic element	CONE (no. of endemics)	List of narrow endemics
Sky islands (4)	Characteristic Endemics: None	**Soutpansberg (2)	*Kerkophorus pumilio*, *Ptilototheca soutpansbergensis*
	Narrow Endemics:	**Wolkberg (2)	*Kerkophorus perfragilis*, *Sheldonia wolkbergensis*
	Four narrow endemics restricted to the Wolkberg & Soutpansberg CONEs		
	Additional species in the same biotic element: None		
**Extended Maputaland (4)	Characteristic Endemics: *Chlamydephorus watsoni*	Middleveld (2)	*Sheldonia monsmaripi*, *Chlamydephorus bruggeni*
	Narrow Endemics:		
	Two narrow endemics restricted to the Northern Middleveld CONE and *Microkerkus sibaya* (restricted to narrow southern Maputaland)		
	Additional species in the same biotic element (shared among the OGUs NMP, SMP, NMV & SMV):		
	*Chlamydephorus lawrencei*		
	*Natalina wesseliana*		
** Natal (6)	Characteristic Endemics: None	Natal Midlands (3)	*Natalina inhluzana*, *Kerkophorus bicolor*, *Microkerkus burnupi*
	Narrow Endemics	Natal Coastal Belt-Ngoye (3)	*Kerkophorus ampliatus*, *K*. *melvilli*, *K*. *russofulgens*
	Six narrow endemics restricted to the Natal Midlands & Natal Coastal Belt-Ngoye CONEs		
	Additional species in the same biotic element: None		
** Extended Pondoland (7)	Characteristic Endemics:	No CONEs are nested within the Extended Pondoland COE	
	*Natalina beyrichi*, and *Kerkophorus vitalis* (widespread in both OGUs of the COE)		
	Narrow Endemics:		
	*Microkerkus pondoensis*, *Kerkophorus puzeyi*, *Kerkophorus terrestris* (restricted distributions in both OGUs of the COE), *Kerkophorus scrobicolus* (restricted to narrow Pondoland OGU) and		
	*Sheldonia fingolandensis* (restricted to narrow southern Transkei coast OGU)		
	Additional species in the same biotic element: None		
** Albany-Knysna (7)	Characteristic Endemics:	Knysna (4)	*Capitina schaerfiae*, *Kerkophorus knysnaensis Nata aequiplicata* (narrowly endemic within the broad Knysna OGU)
	*Afrorhytida kraussi* and *Sheldonia phytostylus* (widespread in the broad Knysna OGU), *S*. *aloicola* and *S*. *hudsoniae* (found in both Albany and Knysna OGUs)		
	Narrow Endemics:		
	*Chlamydephorus parva* restricted to Albany and four species narrowly endemic within the broad Knysna OGU.		
	Additional species in the same biotic element (shared among the OGUs ACB, KNY, SNB & LKR):		
	*Nata watsoni*		
	*Prestonella bowkeri*		
	*S*. *natalensis*		
	*S*. *trotteriana*		

^a^ Districts defined from the UPGMA dendrogram (Fig 2A) with four or more endemic species.

^b^ Geographically contiguous clusters of operational geographic units (OGUs), or individual OGUs in the PAE area cladogram (Fig 4A) with at least two endemic species.

^c^ Biotic elements found by PRABCLUS (Fig 6B).

^d^ Individual OGUs or geographically narrow clusters of OGUs defined by a higher similarity than districts form the UPGMA dendrogram (Fig 2A), harbouring at least two narrow endemic species.

* Characteristic endemics: species occupying more than two-thirds of OGUs in the centre, and/or distributed over a half of the extent of the centre, hence whose range edges roughly coincide with the boundary of the centre; Narrow endemics: species restricted within a single OGU, a CONE or less than half the range of a COE; Additional species in the same biotic element: Species shared among other OGUs indicated for the corresponding biotic element(s) as given in Fig 6.

**Fig 5:**
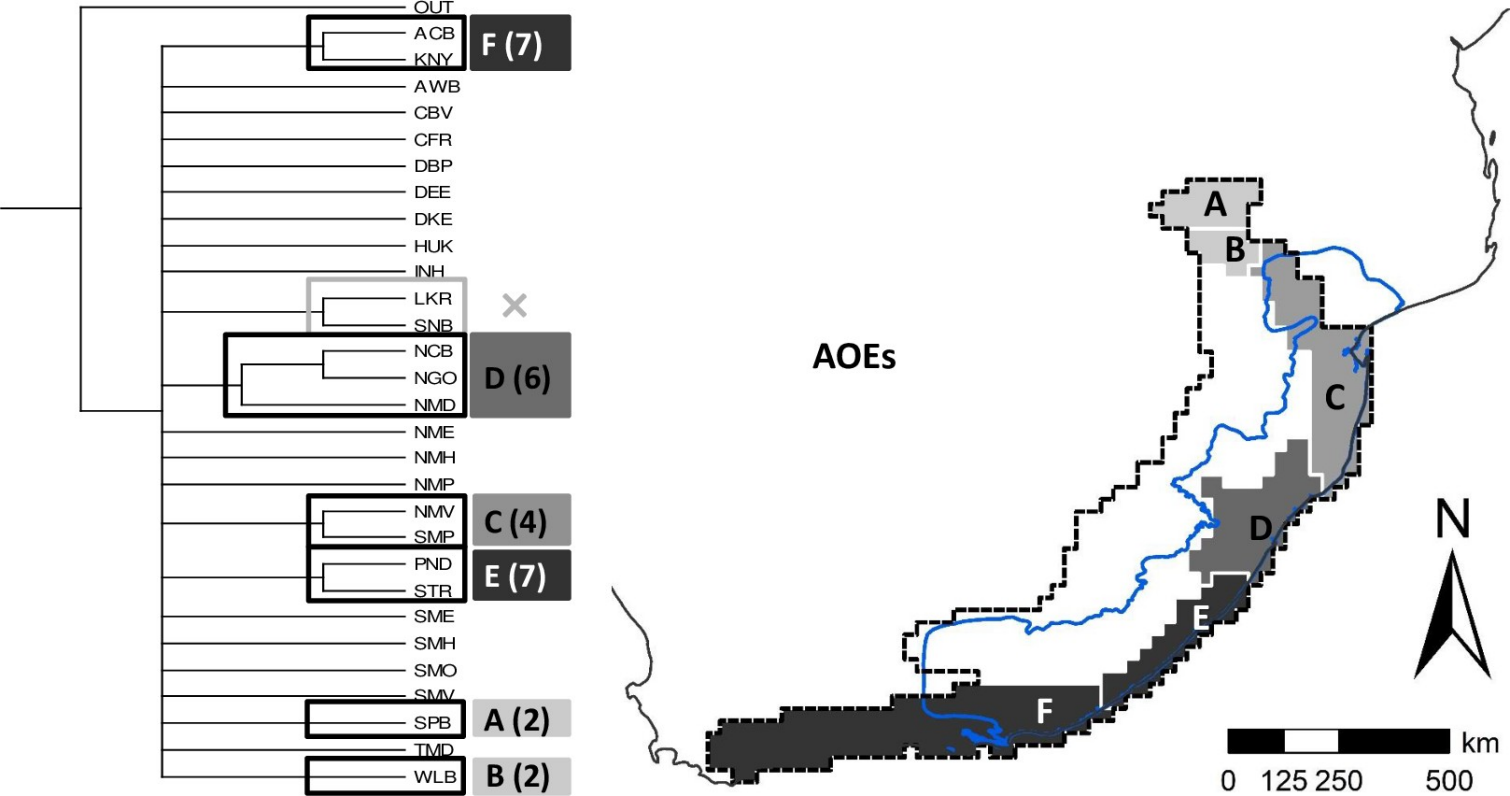
Delineation of areas of endemism (AOEs) for land snails in south-eastern Africa based on the strict consensus tree of parsimony analysis of endemicity (PAE). Four of the five clades in the area cladogram harbour more than two sympatric endemic species, and hence identified as AOEs, together with two OGUs each with two narrow endemics with congruent ranges. All six AOEs are shaded in the map on a grey scale according to the number of endemic species, given below for each in brackets (darker shades denotes high endemism; see Table 3 for lists of endemic species): A—Soutpansberg (2), B—Wolkberg (2), C—Extended Maputaland (4), D—Natal (6), E—Extended Pondoland (7), and F—Albany-Knysna. The MPA hotspot boundary is reprinted from Hoffman et al. [26] (public domain).

Parsimony based AOEs reconfirmed all the COEs derived from phenetic clustering. The consensus tree of the PAE resulted in an area cladogram revealing five clades, among which four harboured at least two endemic species with sympatry, together with two OGUs each with two narrow endemics with congruent ranges. Hence, the PAE identified six AOEs (Fig 5).

**Fig 6:**
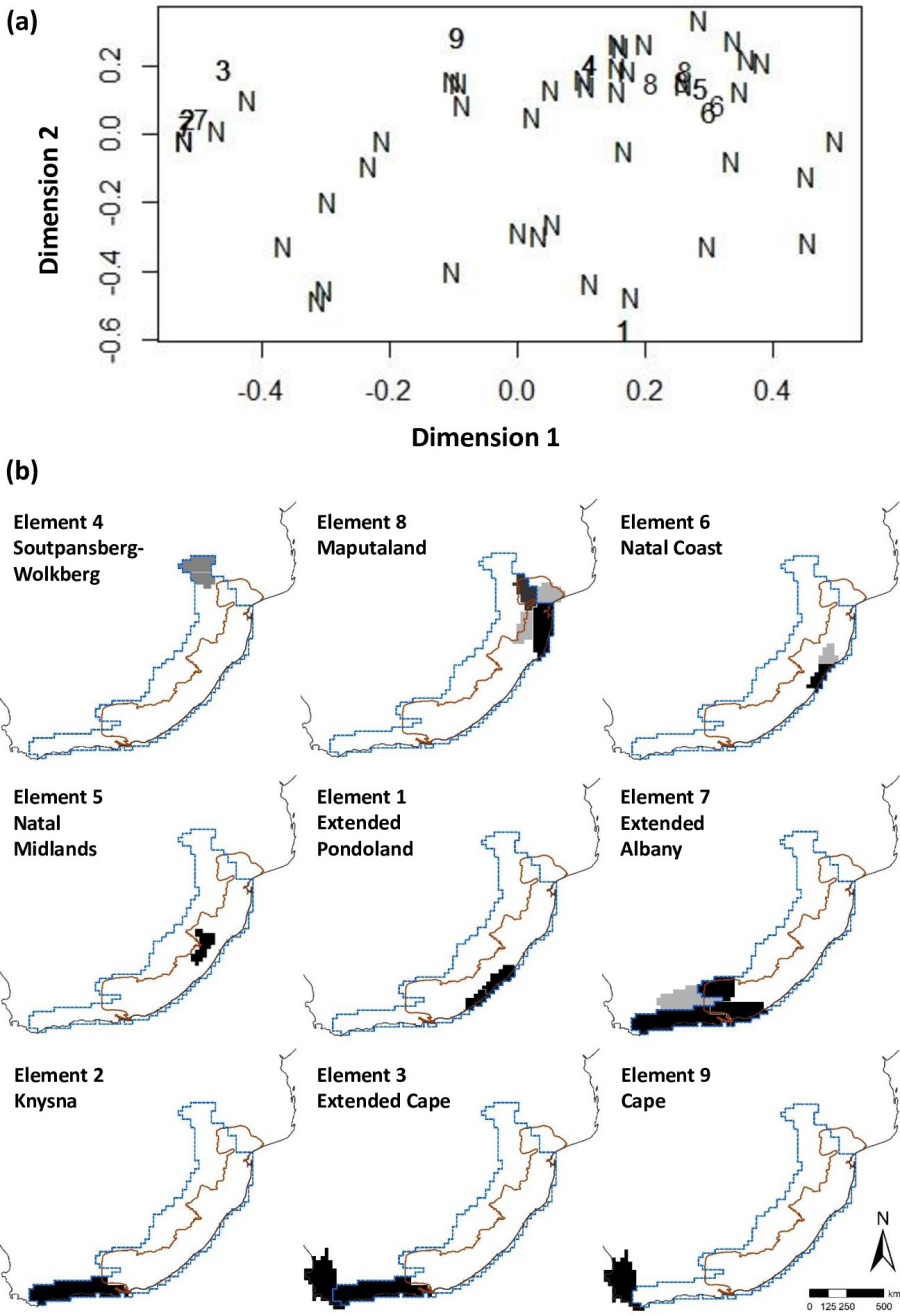
Biotic elements of land snails in south-eastern Africa. **(a)** First two dimensions of the nonmetric multidimensional scaling of the distribution ranges of land snails of south-eastern Africa based on their incidence in pre-defined operational geographic units (Fig 1). Biotic elements found by PRABCLUS are denoted by 1‒9; N = noise component. **(b)** Distribution maps of the nine biotic elements of the land snail fauna of south-eastern Africa. Four different shadings from light to dark indicate the areas where >30%, >45%, >60% and >100% of the species of each element are present. The MPA hotspot boundary is reprinted from Hoffman et al. [26] (public domain).

However, as the PAE area cladogram was not hierarchically well-structured, we further tested our AOEs for consensus and congruency from a BEA. The test for clustering of distribution areas indicated that the distribution ranges of land snails in south-eastern Africa show a clustering tendency (Fig 6A), as the test statistic *t* = 0.429, the ratio between the 25% largest and smallest distances [64, 69] for our dataset, is smaller than expected by the null model (Simulated *t* = 0.451; ranging from 0.355 to 0.627), even though with a low level of statistical significance (p = 0.33). However, when mapped, the BEA yields nine biotic elements, seven of which supporting the six AOEs derived from PAE, and congruent with those five COEs derived from the phenetic clustering. The remaining two biotic elements are supporting another argument we propose later in the present study, i.e. the identification of Knysna as a transitional OGU between the greater MPA region and the greater CFR (already recognised for plants by Born et al. [70]) in land snail biogeography (Fig 6B).

**Fig 7:**
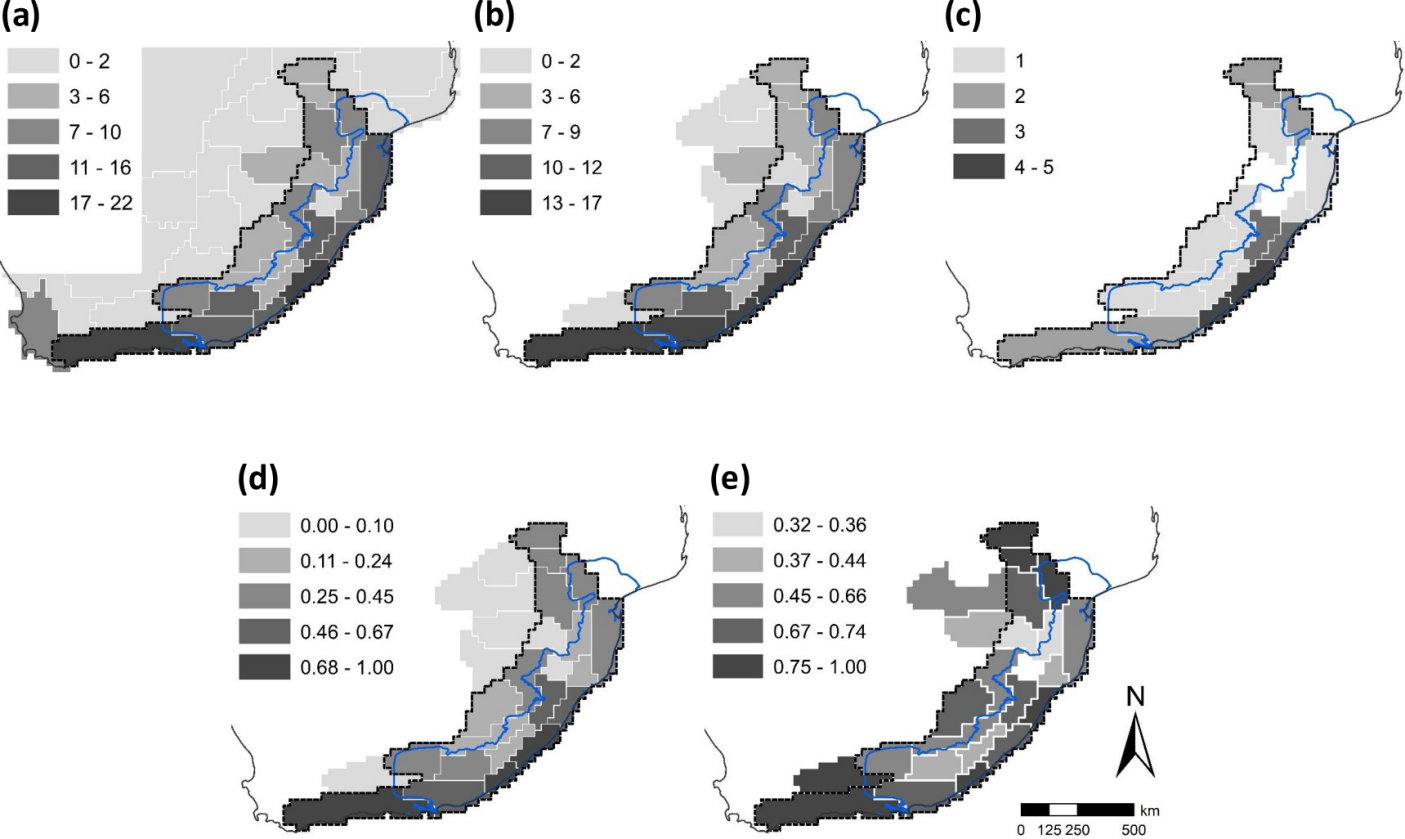
Spatial patterns of land snail fauna in south-eastern Africa (SEA). Species richness for the whole study area and all spatial measures on endemism for the SEA dominion (see Fig 2). **(a)** species richness, **(b)** SEA dominion endemism, **(c)** narrow endemism, **(d)** weighted endemism and **(e)** corrected weighted endemism. See text for details on above calculations. The whole (blue) line indicates the Maputaland-Pondoland-Albany (MPA) hotspot boundary while the dashed (black) line delimits the Greater MPA region of land snail endemism. The MPA hotspot boundary is reprinted from Hoffman et al. [26] (public domain).

Therefore, our COEs for land snails in south-eastern Africa derived from the phenetic cluster dendrogram are supported by both PAE and BEA. The characteristic and narrow endemic taxa of COEs are presented in the Table 3. Twenty-nine species are restricted to COEs, with 21 (72%) of them being narrow endemics. Those species restricted to COEs are dominated by urocyclids (72%) showing an adaptive radiation in narrow ranges (76% of them being narrow endemics) representing almost all centres. They are followed by rhytidids (28%) with one species of *Natalina* each from Natal and extended Pondoland centres, one species each of *Afrorhytida*, and *Nata* from the Albany-Knysna centre, one species of *Capitina* from the Knysna CONE and one species of *Chlamydephorus* each from the Albany-Knysna centre and the Middleveld CONE. A spatial comparison of COEs with biomes [25] and the protected area network of SA is presented in Fig 4D and 4E. Spatial patterns of land snail species richness for SEA and measures of endemism within the SEA dominion are mapped in Fig 7.

### A greater Maputaland-Pondoland-Albany region of land snail endemism

The COEs as well as the patterns of land snail endemism confirm the hotspot status of MPA for malacofauna (see Figs 4–7). Furthermore, the MPA-Knysna province (Fig 2) is recognized as a numerically derived zoogeographical entity for land snails. Nevertheless, the land snail endemism as indicated by the COEs (Fig 4) and high levels of weighted endemism (Fig 7), extends beyond this province towards the south-eastern escarpment (Drakensberg-KwaZulu-Natal escarpment, Drakensberg plateau, and Sneeuberg OGUs) and the eastern escarpment (northern & southern Mpumalanga escarpment, Wolkberg and Soutpansberg OGUs), comprising many OGUs recently recognised as individual centres of endemism [29, 71, 72]. The incorporation of these OGUs into the MPA-Knysna province designates the greater MPA region of land snail endemism (see Figs 4–7), with 57 endemic species (82.6% endemicity; Table 2) from selected land snail genera. The greater MPA region for malacofauna, being 46.7% larger in area compared to the MPA hotspot, harbours a remarkable 78.1% greater number of endemic land snail species (57 endemic species in the greater MPA region compared to only 32 in the MPA hotspot; Fig 3). Moreover, spatial pattern of species richness further justifies the greater MPA region with OGUs such as southern Maputaland, Natal coastal belt, Natal Midlands, Pondoland, southern Transkei coastal belt, Albany coastal belt, Amatola-Winterberg and the Knysna transition zone (from North to South) been identified as hotspots of high species richness, while northern Mpumalanga escarpment, northern Middleveld, Ngoye, Drakensberg-KwaZulu-Natal escarpment and the Sneeuberg are also fairly speciose in land snails (Fig 7A).

## Discussion

### Land snail biogeography of south-eastern Africa

The study provides a preliminary assessment of the land snail biogeography in SEA, based on a selection of the most well-known genera. The delineation of the SEA dominion separated from the Cape in south-west, Lowveld in north-east and especially the Karoo and Kalahari in the west is driven mainly by the climatic factors separating xeric areas from relatively mesic ones, as evident in previous regionalisations for vertebrates [24, 45, 57, 73–76], higher plants [45] and bryophytes [77].

The subsequent separation of the MPA-Knysna province from more inland OGUs seems to have ecological as well as historical explanations related to the greater habitat heterogeneity along the coastal belt and the cyclical expansion and contraction of forests over geological time [57, 78–80], contrasting with the more homogeneous savanna in Bushveld province, grasslands in Highveld province, and the lower Karoo in the southern escarpment subdominion. The refugial role played by the patchy distribution of forests during the Pleistocene climatic cycles with corresponding expansions and retractions [78, 80, 81] are presumed to have shaped the present-day malacofauna of the area (see Fig 4D). The repeated isolation of once linked forest patches may have contributed to rapid diversification and increased narrow endemism [82], also leaving ancient Gondwanan geographical relicts in isolated forest patches due to the relative stability in climate within them [13, 34, 83]. The role of ecological stability of forest habitats in promoting radiation of relictual taxa has been recognised [84], while this phenomenon has also been documented for land snails in forest refugia along the Albertine rift valley of Africa [85], which can be attributed to the ecophysiology of land snails, including their preference for habitats with high micro-habitat complexity, high humidity, low temperature and damp soil with decaying organic matter [86–88].

The southern escarpment subdominion, possibly a partial recovery in the present analysis corresponds to the lower Karoo bioregion. The MPA-Knysna province indicates an extension of MPA elements into the Cape, which is distinct from the original delimitation of the MPA hotspot. The Knysna unit spatially represents the eastern section of the CFR biodiversity hotspot, characterized by the Fynbos biome. The similarities of the malacofauna of the Knysna OGU (eastern CFR) with further eastward OGUs are associated with the presence of forest patches in them, despite the Knysna OGU being dominated by Fynbos. This can be attributed to the post-glacial expansion of Afromontane forests to the Knysna-Tsitsikamma area [80, 89]. Overall similarities in the present regionalisation to that for vertebrates [24], despite fine scale differences likely caused by life history attributes such as body size and dispersal ability [90], suggest a common zoogeographical pattern for both invertebrates and vertebrates in SEA, while biogeographic patterns available for plant assemblages in southern Africa also support similarities [91]. This opens up avenues for future studies incorporating more invertebrate taxa, where there is adequate spatial data and a sufficiently robust taxonomy, and for cross taxon analyses involving both a wide suite of animal and plant lineages. Furthermore, the consensus for an extension of the MPA into the Knysna OGU in both vertebrate and land snail regionalisations also suggest further investigations on the limits of a Knysna transition zone between the greater CFR and the greater MPA region. Concerning land snails, the proposed regionalisation permits further refinement when a more complete data set is available at a finer scale equal-area geographical unit, for more genera, to support a much more rigorous analysis, while the data becoming newly available supports the patterns identified here (e.g. [92, 93]).

### Land snail endemism in south-eastern Africa

Quantitative measures mapped for land snail species richness and endemism reveal similar patterns (Fig 5), with higher values along the south-eastern coastal belt and on the south-eastern escarpment, especially its eastern aspects towards the coastal belt, similar to those for millipedes [94, 95], vertebrates [24], bryophytes [96] and higher plants [20]. The Highveld and Bushveld provinces are generally species- as well as endemic-poor, although the Bushveld supports more land snail endemics compared to the Highveld (Fig 5E), which could possibly be attributed to the comparatively higher habitat heterogeneity in the Bushveld that provide sheltering microhabitats into which snails can retreat, supported by the higher spatial area covered by Bushveld, and again to the supposedly stable and milder climate in the Bushveld (see the role of forests discussed above) compared to that of the Highveld characterized by drought, frost, waterlogging and wildfire causing its treelessness [97].

The northern Mpumalanga escarpment, northern Middleveld, southern Maputaland, Ngoye, Natal coastal belt, Natal Midlands, Drakensberg-KwaZulu-Natal escarpment, Pondoland, southern Transkei coastal belt, Amatola-Winterberg, Albany coastal belt and Knysna OGUs (from North to South) are established as strongholds of both species richness and endemism of land snails (Fig 5A and 5B), while narrow endemism is concentrated within the extended Pondoland and Natal districts (Fig 5C). Measures of weighted endemism (Fig 5D) further emphasize the importance of the land snail districts of Soutpansberg-Wolkberg, northern Middleveld, southern Maputaland, Drakensberg-KwaZulu-Natal escarpment, Natal, extended Pondoland, and Albany-Knysna (from North to South) for malacofaunal conservation, strongly supporting the concept of a greater MPA region of land snail endemism. The corrected weighted endemism (Fig 5E) further highlights the Drakensberg plateau and the lower Karoo as important for land snail conservation in addition to the above districts. Recovery of similar endemism peaks from both the UPGMA and PAE clustering as well as the BEA re-confirms the importance of the greater MPA region for restricted-range land snail radiations.

Pondoland, a prominent centre of plant endemism, is well recovered here for land snails, even though it showed a comparatively lower endemicity for vertebrates [24]. Nevertheless, the Natal centre, well established for vertebrates, was also found to be an important centre for land snails, even though it is not widely acknowledged for plants. In contrast to the western limits of the Albany COE for flora [20], land snail endemism in this area extends westward recovering a single Albany-Knysna COE. The overlap of the distribution of forest and Albany thicket biomes with COEs for land-snails (Fig 4D) is noteworthy for further investigation, particularly at a finer scale, while the present study confirms a congruence of endemism between plants and land snails.

### The data revisited

The species selected comprise a representative sample of endemic land snail lineages of SEA and their distributions collectively span much of the study area. More importantly, the land snail genera selected for the study are some of the best collected throughout the study area and with a recently well-resolved taxonomy. We did not source other museum databases except for the KwaZulu-Natal Museum, since it represents by far the largest collection of land snails in SA. Furthermore, the KwaZulu-Natal Museum database also includes the locality data from the monograph of South African non-marine Mollusca [98] and thus overlaps extensively with the holdings of the South African Museum for the selected genera. There is also a considerable overlap with the East London Museum collection through a collaborative field work programme spanning the last 20 years. Hence, the inclusion of other smaller collections is unlikely to change the species incidence matrix for the selected genera at the coarse scale of OGUs used for this study.

Although less likely, any errors in the boundaries of proposed land snail biogeographical entities, owing to qualitatively derived vertebrate-based boundaries of OGUs, could only be corrected once the land snail incidence data are available at an appropriate level of completeness to facilitate an analysis at a finer equal-area grain, preferably at the QDS scale. Unfortunately, land snail species have not been sampled intensively at QDS scale in SA [15], and poor or spatially heterogeneous sampling can result in unreliable biogeographical regionalisation patterns [99].

### Consensus across methods on patterns of endemism

The primary objective of our analyses was to attempt a land snail-based bio-regionalisation of the area and thence to see if that would support the results of a similar analysis conducted on vertebrates [22, 24]. Standard methods endorsed by Kreft & Jetz [39] for biogeographical regionalisation, which have been followed by the majority of subsequent studies throughout the world were used for the main analysis of our research, i.e. agglomerative hierarchical clustering with UPGMA algorithm. Using this method, we regionalised the area and further identified the geographic clusters of the dendrogram with an aggregation of range overlapping endemic species as COEs.

However, we further attempted establishing the AOEs using PAE described by Morrone [59], being the most widely used method for the identification of AOEs (see Morrone [100] for a detailed discussion). PAE is designed for incidence data matrices of equal area grid cells and it performs best using such OGUs. Our data matrix uses pre-defined eco-geographical units as OGUs, and hence several equal area grid cells with ecological similarity are already combined in a single OGU, possibly lowering the power of PAE to define the complete hierarchy of AOEs, as it makes it difficult to numerically establish the strict sympatry of taxa, however still being able to define AOEs based on the clades in area cladogram. Perera et al. [22] described the delimitation of our OGUs through a qualitative overlaying of range maps in identification of endemic vertebrate distributions (= biochoria [70] for endemic vertebrates), which are biogeographical area units other than AOEs [101, 102]. Therefore, using such units here for identification of AOEs of land snails is not circular and warranted. The determination of AOEs was the prime objective of carrying out a PAE in our study, while those identified AOEs are perfectly congruent to COEs identified from the UPGMA clustering. PAE has exclusively been used in regionalisation attempts to provide hierarchical area cladograms of geographic units in some global to sub-continental scale studies on entire faunas [24, 60, 61], in which more endemics are captured, increasing the power of the analysis. However, we believe the power of PAE in doing so depends on several factors such as (a) higher numbers of endemic taxa in the matrix, (b) higher number of OGUs being occupied by more than one species, and (c) whether the study uses equal area OGUs defined by a grid of squares. Our OGUs were not equal area units nor were they square in shape, while the matrix had only 73 species with 15 out of 40 OGUs being occupied only by a single or no species (parsimony uninformative), thence resulting in a low level of hierarchical structuring in the PAE area cladogram. Hence, with a restricted spatial extent and taxonomic coverage in our study, we did not attempt a regionalisation exclusively based on PAE as done in previously mentioned studies. However, less structuring in our PAE area cladogram does not necessarily negate the hierarchical structure suggested by the UPGMA clustering, as it depends only on the similarity coefficients between each of the OGUs based on entire species (either endemics or not) assemblages. Consequently, we acknowledge that the present dataset indicates limitations in matching the hierarchical outputs from PAE and UPGMA clustering approaches.

However, in order to test the robustness of endemicity findings, we performed a BEA [63], including a parametric bootstrap test for clustering of distribution areas [64]. Similar to PAE, the BEA has also been designed to be used in grid-based equal area OGUs and hence have a lower power in detecting the structuring in our OGUs, as OGUs with varied shapes result in a neighbourhood matrix with uneven numbers of neighbouring units. However, the BEA indicated our data also show a certain degree of clustering (with a test statistic lower than expected by the null model, although with a lower level of statistical significance (p = 0.33)). In evidence to the diminished power of BEA when using non-grid based OGUs, our statistical significance was increased to (p = 0.21), by adjusting our neighbourhood data considering only four neighbouring OGUs for a given unit. Our argument can be further supported by Hausdorf & Hennig’s [63] observation that the same dataset when used at different scales clustered well at the 2° grid scale, but not at a finer 1° grid scale.

This illustrates one of the biggest issues in numerical biogeographical analysis, i.e. although the use of equal area grid cells is recommended as the standard practice, it is accompanied by trade-offs of data completeness at fine scales, and distortion of natural boundaries and conglomeration of habitat heterogeneity (especially crucial if patchy habitats are important for the study lineage) at large scales of equal area grid cells. However, Hennig & Hausdorf [103] proposes an approach to be used under such issues of data incompleteness at an appropriate scale of equal area grid cells, which is unsuitable for our data matrix with uneven OGUs.

Our study marks (a) the first ever analysis on the biogeography of land snails in south-eastern Africa and (b) the first attempt to study the biogeography of an invertebrate group of the area using UPGMA based phenetic hierarchical clustering of OGUs. However, an incidence data matrix for the weevil genus *Sciobius*, at 2° grid squares for the same study area has been used by Morrone [59] to introduce and substantiate the application of PAE. Several more studies have used the same dataset to conduct various numerical biogeographic analyses and compare their results, viz. Szumik et al. [104] introducing an optimality criterion for AOE identification; Mast & Nyffeler [105] proposing the use of a null model to recognise significant co-occurrence of taxa; Hausdorf & Hennig [63] introducing BEA also using a null model to test for clustering; Hennig & Hausdorf [103] to account for incomplete sampling in BEA and Escalante [106] comparing PAE with Analysis of Endemicity. Therefore, we not only analysed our dataset using the PAE and BEA in addition to the UPGMA clustering of OGUs, but also checked for cross taxon congruence of our land snail AOEs with those for *Sciobius* weevils.

Our biotic elements support all five COEs of land snails in south-eastern Africa including those six AOEs derived from PAE for the same data set. Furthermore, our land snail COEs and AOEs correspond well with the AOEs and biotic elements of other studies [59, 63, 104–106] derived for a 2° equal area OGU based dataset of *Sciobius* weevils in south-eastern Africa as summarised in the Table 4. Additionally, two of our biotic elements (elements 7 and 2) support the Knysna extension of the proposed greater MPA region, while two more (elements 9 and 3) supports the argument of the Knysna transition zone between the greater MPA province proposed here and the greater CFR delimited by Born et al. [70].

**Table 4:** Comparison of centres and areas of land snail endemism in south-eastern Africa (SEA), defined in the present study through UPGMA clustering, PAE and BEA with similar areas defined for *Sciobius* weevils of south-eastern Africa using various methods employed in previous studies.

Reference	Taxa	Units	Method	Comparison of Results				
Present study	Land snails of SEA	COEs	UPGMA	Sky islands	Extended Maputaland	Natal	Extended Pondoland	Albany-Knysna
		AOEs	PAE	Soutpansberg	Extended Maputaland	Natal	Extended Pondoland	Albany-Knysna
				Wolkberg				
		BEs	BEA	Element 4	Element 8	Elements 5 & 6	Element 1	Elements 2 & 7
Morrone [59]	*Sciobius* weevils of SEA	AOEs	PAE	-	Area 1 (partly)	Area 1	Area 3	Area 2
Szumik et al. [104]		AOEs	AE	Sets 10 & 11 (partly)	Sets 2, 10 & 11	Sets 1, 4, 5, 6, 7, 8 & 9	Set 7	Sets 3, 12, 13 & 14
Mast & Nyffeler [105]		AOEs	PAE	-	Area 1 (partly)	Areas 1	Area 3	Area 2
Hausdorf & Hennig [63]		BEs	BEA	Element 1 (partly)	Element 1	Element 2	Element 4	Element 3
Escalante [106]		AOEs	AE	Set 2 (partly)	Set 2	Sets 0, 2 & 3	Set 3	Set 1

(UPGMA—phenetic hierarchical clustering with UPGMA algorithm with the Jaccard index; PAE–Parsimony analysis of endemicity; BEA–Biotic element analysis; AE—Analysis of Endemicity; BEs–Biotic Elements).

A few OGUs such as Drakensberg-Eastern Cape escarpment, northern Natal, southern Middleveld, Waterberg, Highveld-upper Karoo and Inhambane that were inappropriately placed in the cluster dendrogram with comparatively low species richness and endemism may indicate gaps in biogeographic knowledge or more likely artefacts of sampling biases. Northern Natal and Waterberg have recorded none of the selected species while only two species have been recorded from Highveld-upper Karoo, which may indicate gaps in sampling. It is apparent that those areas need prioritised attention with respect to malacofaunal inventorying surveys.

The northern Natal and Transkei Midlands (adjacent to Drakensberg-Eastern Cape escarpment) were also recognised as gaps for vertebrate endemism [22], the latter supporting the idea of a Transkei Gap [107]. However, even if such a real biogeographical gap exists for land snails along the Transkei Midlands/escarpment, it certainly does not extend to the coastal belt, instead identifying the extended Pondoland as one of the most prominent COEs (see Figs 4 and 5).

### The MPA hotspot, the greater MPA region of endemism and conservation implications

While the hotspot status of the MPA is further confirmed by its malacofauna, the greater MPA region provides a zoogeographically delimited broader region of land snail endemism with five COEs serving as local conservation priorities. A remarkable 72% of the species restricted to COEs are narrow endemics, highlighting the habitat heterogeneity provided within the greater MPA region of endemism. Endemism-based intuitive malacofaunal subregions presented for eastern SA [13] captured similar (if not finer) geographical entities congruent with our Natal and extended Pondoland COEs as well as an eastern escarpment (sections of the great escarpment in Mpumalanga and Swaziland) extension, further supporting the greater MPA region of land snail endemism. Further, the six AOEs derived from PAE and the nine biotic elements yielded from BEA are congruent to those five COEs, well supporting our proposals for the greater MPA region of land snail endemism and the Knysna transition zone between the greater CFR and the greater MPA region.

The greater MPA region for land snails is much similar to that recognized for vertebrates [24], than to the more restricted MPA hotspot. This suggests the possibility of the greater MPA being a common region of animal endemism–one that needs to be tested for cross-taxon congruence with other invertebrate groups. The current vertebrate bias in conservation planning needs to be addressed as more data become available for invertebrate groups, which can substantially broaden the taxonomic basis for conservation prioritization [9, 108–111]. Since many invertebrate groups have limited vagility, they also facilitate the identification of CONEs, which might be overlooked in an analysis of the flora or vertebrate fauna at the same scale. A preliminary spatial comparison of CONEs with existing protected areas reveals a major gap in Pondoland and the southern Transkei coastal belt (Fig 4E), where the land snail endemism is highest (Fig 5), while Natal Midlands, Natal coastal belt-Ngoye, northern Mpumalanga Escarpment and Soutpansberg are also not adequately protected. Hence, as acknowledged previously [15], the protection of CONEs identified here needs urgent attention. Mechanisms for the protection of smaller parcels of land targeted at the conservation of narrow endemic and less vagile invertebrates need to be included into conservation planning.

## Supporting information

S1 Appendix Land snail species incidence matrix (n = 73; 12 genera and three families) in 40 operational geographic units (OGUs). See Fig 1 for the OGU codes.
(XLSX)
